# Supplementary figures and images for: Interleukin-27-induced HIV-resistant dendritic cells suppress reveres transcription following virus entry in an SPTBN1, autophagy, and YB-1 independent manner
Source: PLoS One. 2023 Nov 1;18(11):e0287829. doi: 10.1371/journal.pone.0287829 (PMC10619827; doi:10.1371/journal.pone.0287829)

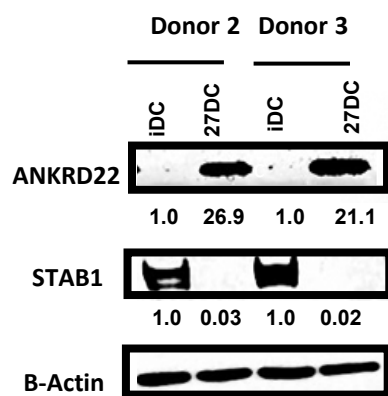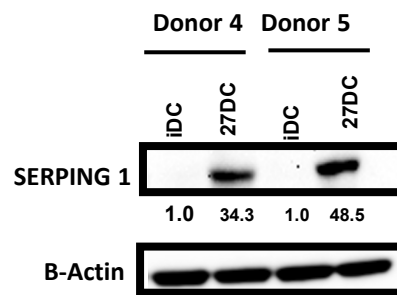

Supplement: S1 Fig — The total cell lysate was collected from iDC and 27DC of three different donors, and Western blotting was performed using anti-ANKRD22, anti-STAB1, and anti-beta Actin antibodies. The band intensity of each protein was normalized by the band intensity of GAPDH (Image J); the values are indicated below images. (PDF) [file pone.0287829.s001.pdf]

(A)

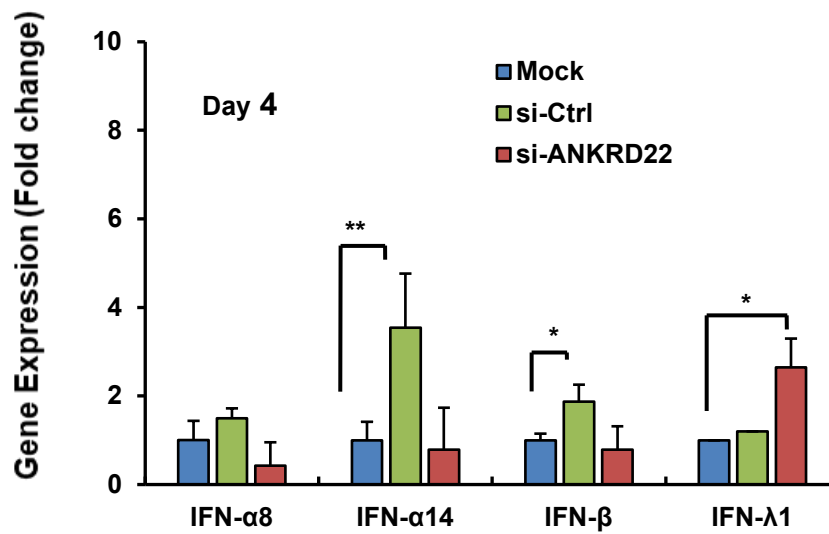

(B)

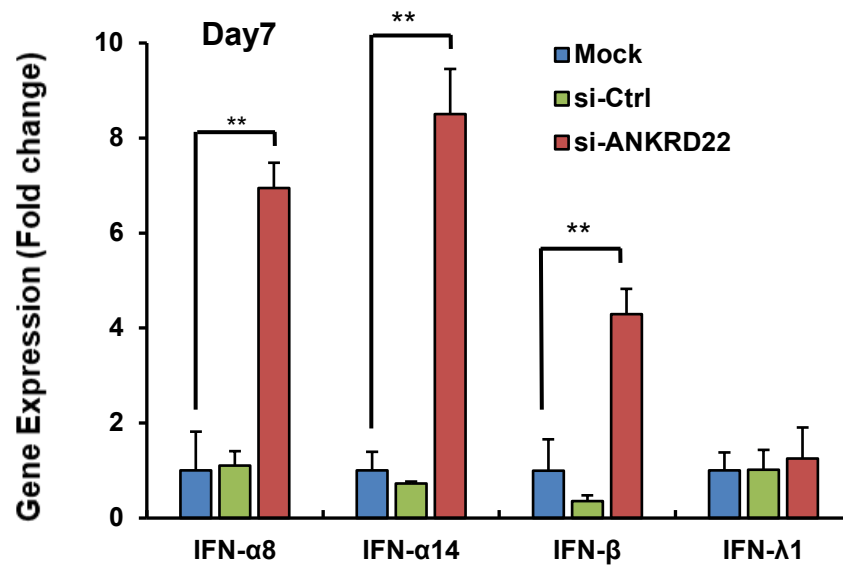

Supplement: S2 Fig — Monocytes (1x10^7^ cells) were transfected with si-Ctrl or si-ANKRD22 using HiPerFect (Qiagen) as described in the Materials and Methods. As a control, monocytes were mock treated with HiPerFect alone. The transfected cells were cultured for 7 days in G4-media. The cells were collected for RNA extraction on day-4 and day-7. Relative IFN-α8, IFN-α14, IFN-β, or IFN-λ1 expression was measured by qRT-PCR. two independent assay was performed, and one of the representative dates is shown. Data are shown as mean ± SD (n = 3), and the gene expression level was compared to mock-treated cells. ** p,0.01, ***p< 0.001. (PDF) [file pone.0287829.s002.pdf]

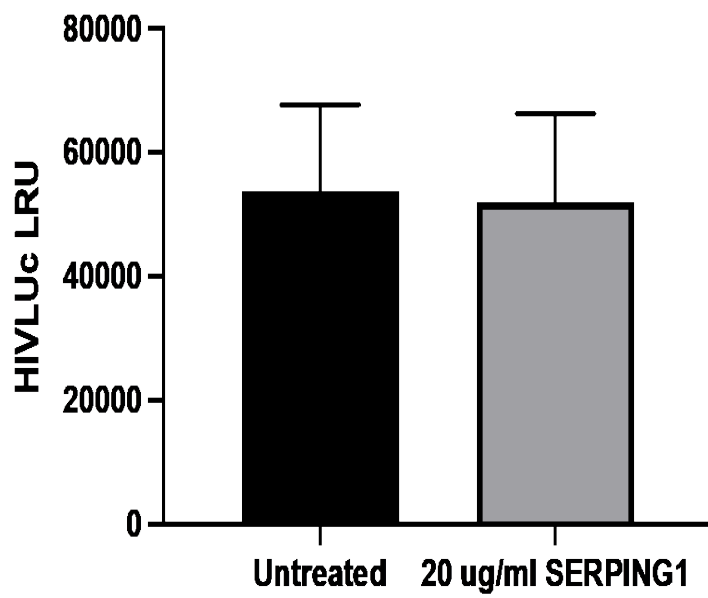

Supplement: S3 Fig — iDC were cultured with 20 μg/mL of recombinant SERPING1 for 3 days and then infected with HIVLuc as described in the Materials and Methods. HIV infection was quantified by luciferase assay. Data show Mean ± SD (n = 3) of a representative data from two independent assays. (PDF) [file pone.0287829.s003.pdf]
