# Supplementary material for: Interleukin-27-induced HIV-resistant dendritic cells suppress reveres transcription following virus entry in an SPTBN1, autophagy, and YB-1 independent manner
Source: PLoS One. 2023 Nov 1;18(11):e0287829. doi: 10.1371/journal.pone.0287829 (PMC10619827; doi:10.1371/journal.pone.0287829)
Supplement: S1 Table — Differentially expressed gene candidates were selected for verification with an absolute fold change difference greater than 3.0 with significant differences (p<0.05) determined by two-way ANOVA between iDC and 27DC in each donor. Total 51 genes, commonly either up- or downregulated in four donors were selected by Venn diagram analysis. (PDF) [file pone.0287829.s004.pdf]

Supplementary Table S1

Gene list commonly changed 3 fold in 27DC ($p<0.05$)

#	LHRI >3 in 4 donor	p value
1	MIR4524A	0.00837
2	IL17RB	0.04980
3	CCL18	0.03337
4	CCL23	0.03668
5	ABCA6	0.02139
6	SLC40A1	0.02433
7	ADAM23	0.00451
8	CD163L1	0.01728
9	GPR171	0.03794
10	FOLR2	0.04334
11	S1PR1	0.03785
12	LOC730139	0.00103
13	RN5S51	0.01330
14	TMEM71	0.03932
15	SLAMF1	0.04996
16	AIG1	0.03441
17	PTGER3	0.03199
18	HRH1	0.02869
19	HS3ST2	0.04442
20	FAM190A	0.03968
21	ZNF480	0.04869
22	EHF	0.03240
23	TOX	0.04085
24	CCR6	0.03003
25	ZNF827	0.00299
26	GIMAP4	0.00859
27	HCP5	0.00015
28	CTLA4	0.02386
29	CDA	0.01549
30	TLR3	0.01324
31	IFIT3	0.03686
32	FGD5	0.00194
33	INHBA	0.01114
34	FLT1	0.01719
35	GBP2	0.00147
36	IRF1	0.01186
37	GBP2	0.00266
38	IFI6	0.00075

39	CLEC4D	0.00349
40	MYOF	0.00116
41	CLEC5A	0.00479
42	LGALS3BP	0.00237
43	TNFSF10	0.01349
44	ANKRD22	0.04972
45	PLD4	0.01016
46	GBP1	0.00147
47	GBP4	0.00794
48	GBP5	0.00687
49	CLEC6A	0.00591
50	SERPING1	0.00124
51	STAB1	0.02613
