# Supplementary material for: Interleukin-27-induced HIV-resistant dendritic cells suppress reveres transcription following virus entry in an SPTBN1, autophagy, and YB-1 independent manner
Source: PLoS One. 2023 Nov 1;18(11):e0287829. doi: 10.1371/journal.pone.0287829 (PMC10619827; doi:10.1371/journal.pone.0287829)
Supplement: S1 Raw images — (PDF) [file pone.0287829.s005.pdf]

Fig. 4A

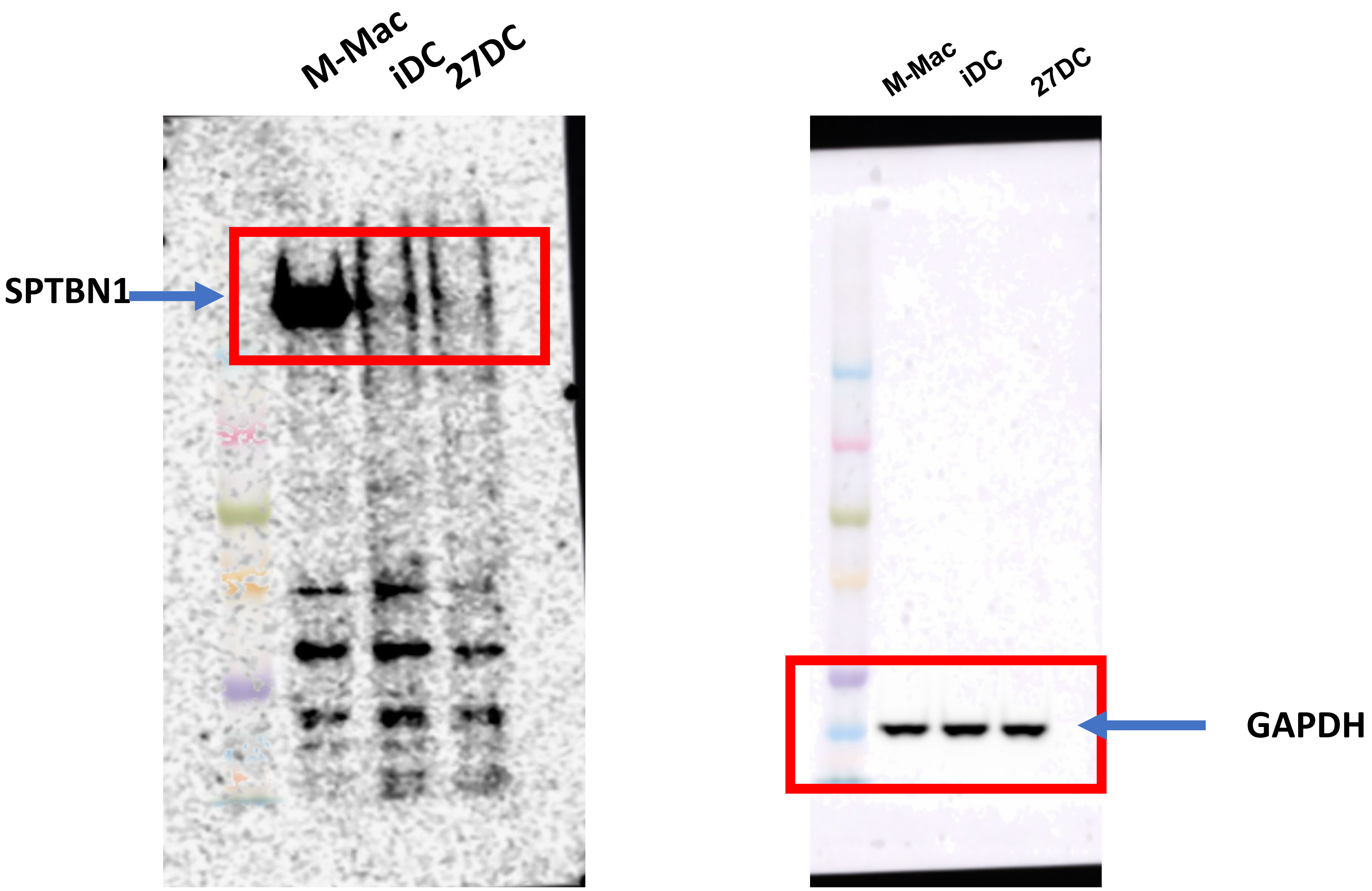

**Fig. 4B**

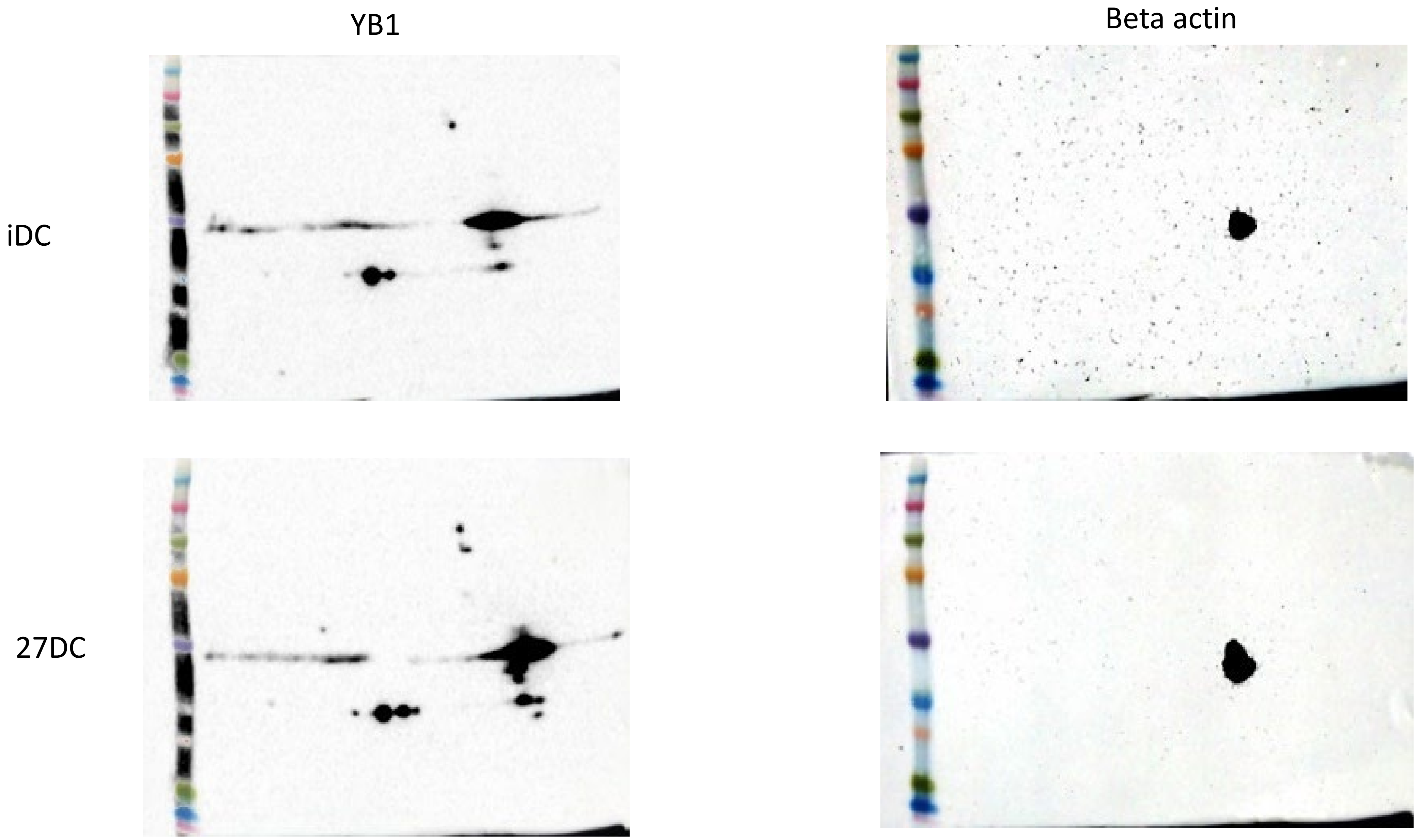

Fig. 5C

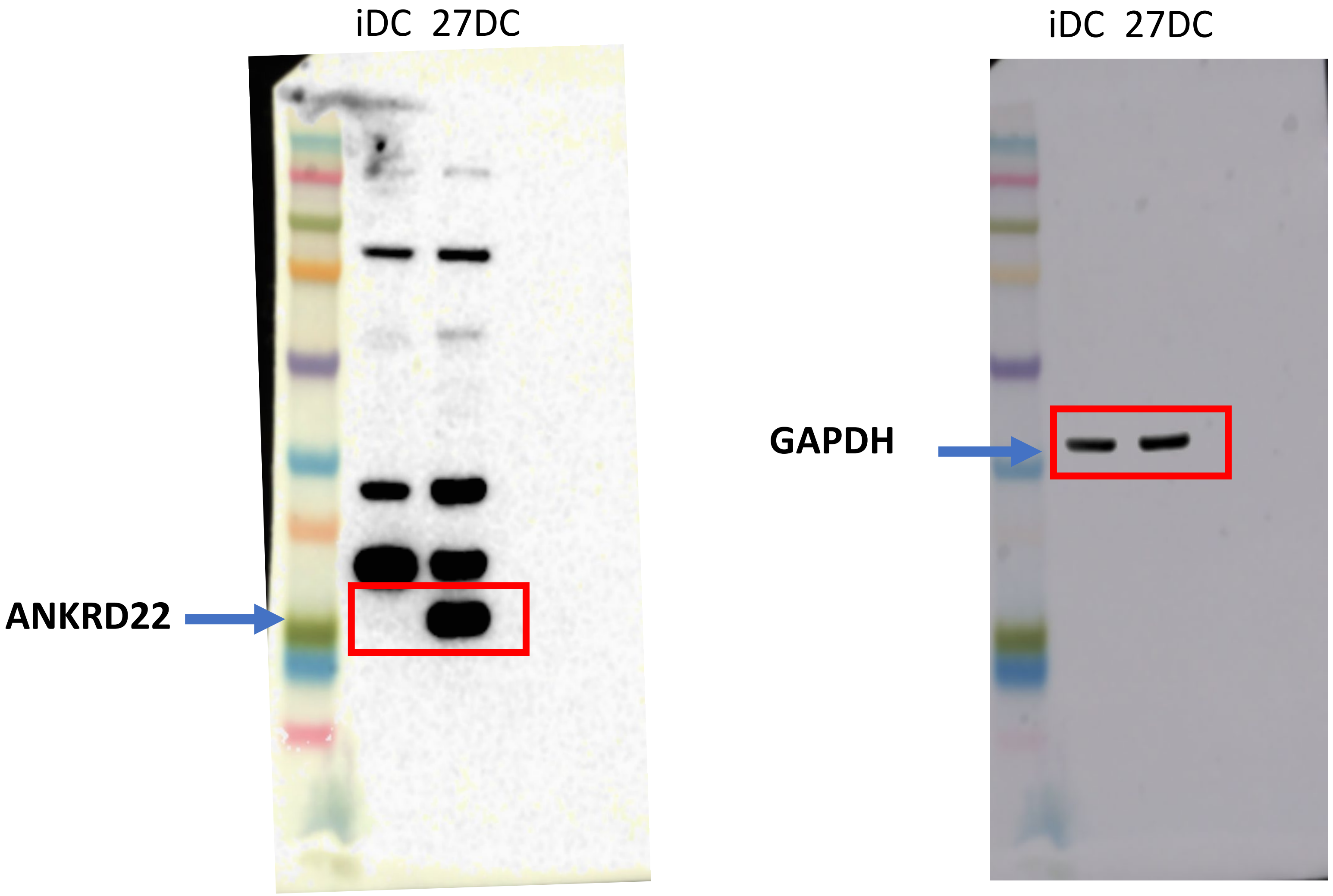


Red box area are used in the figure

Fig. 5C

Red box area are used in the figure

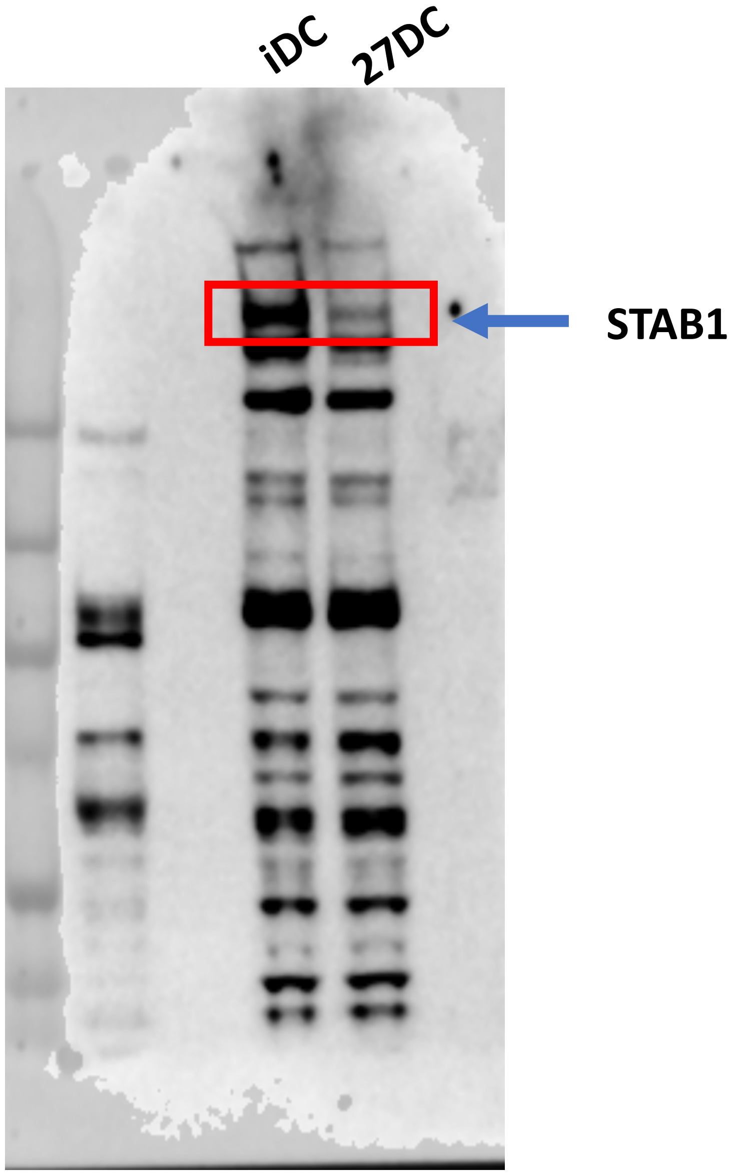


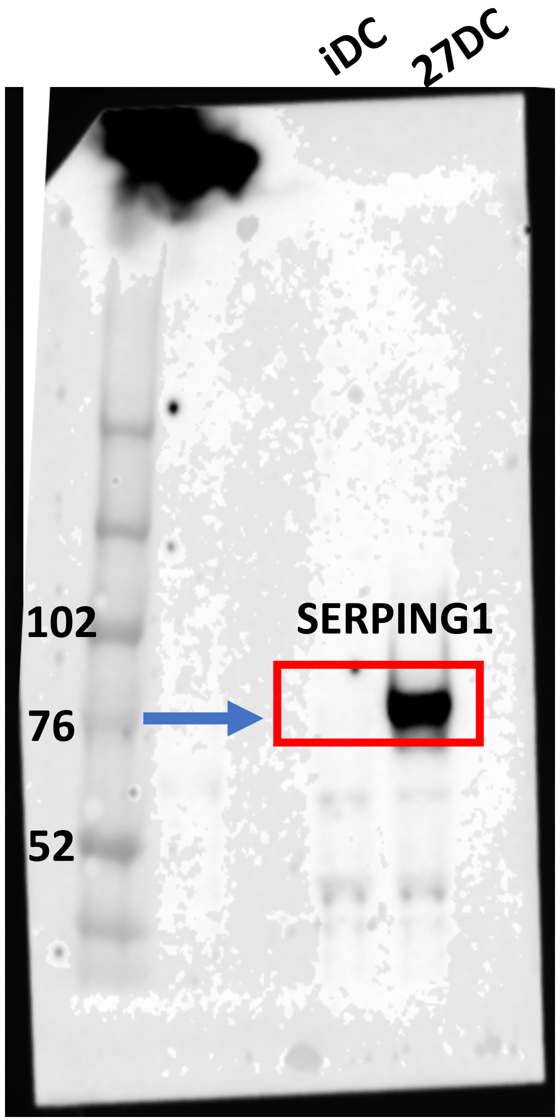

Fig. 6A

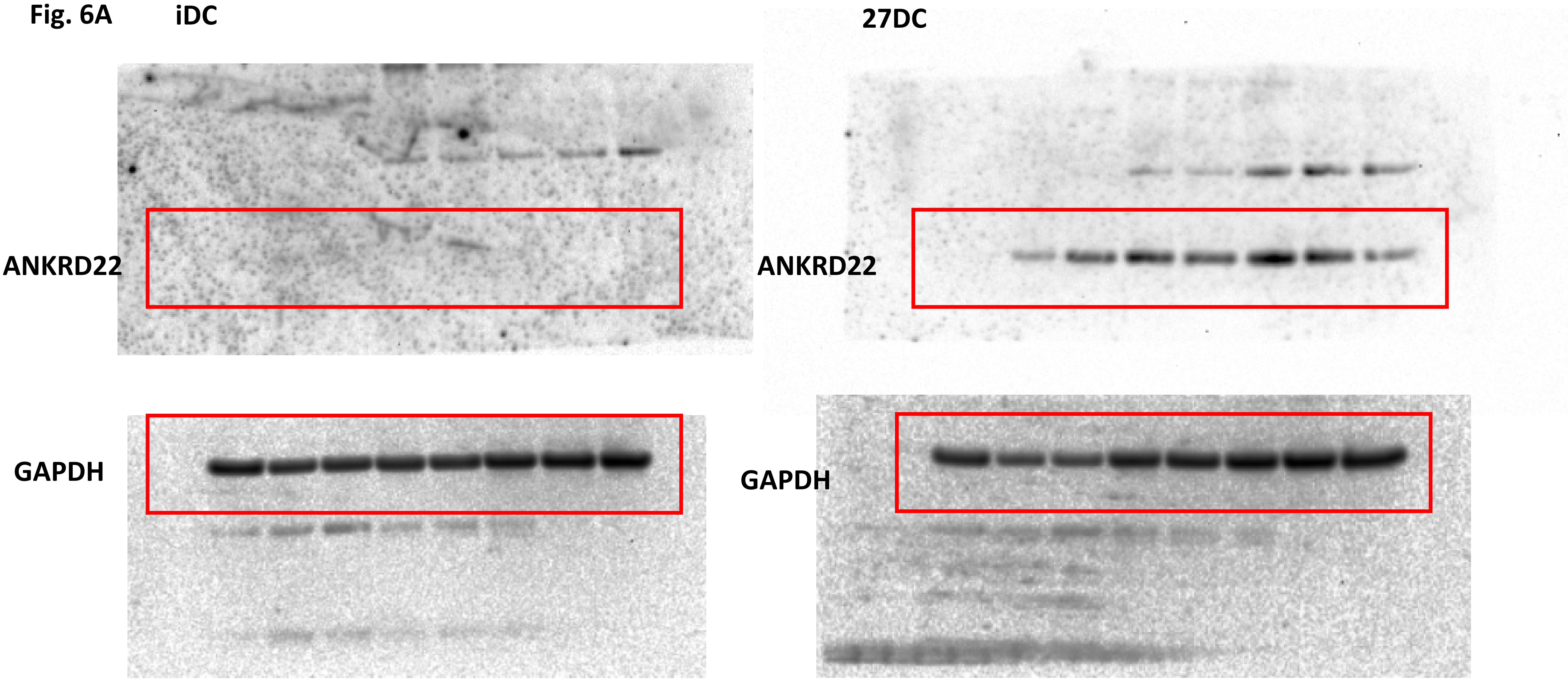


Red box area are used in the figure

Fig. 6C

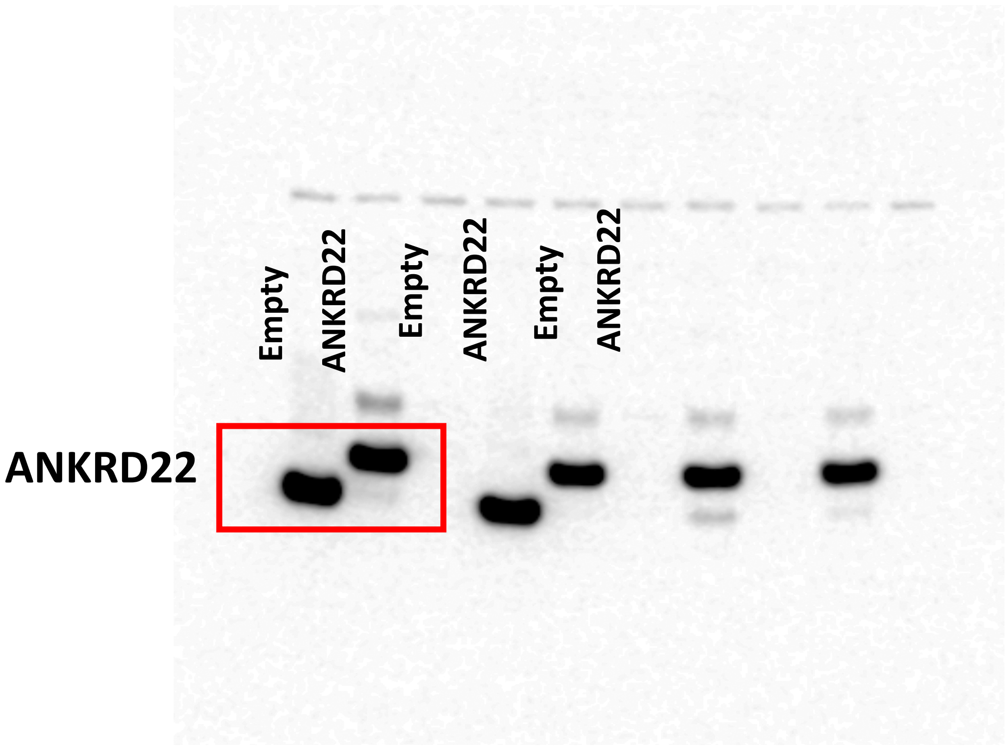


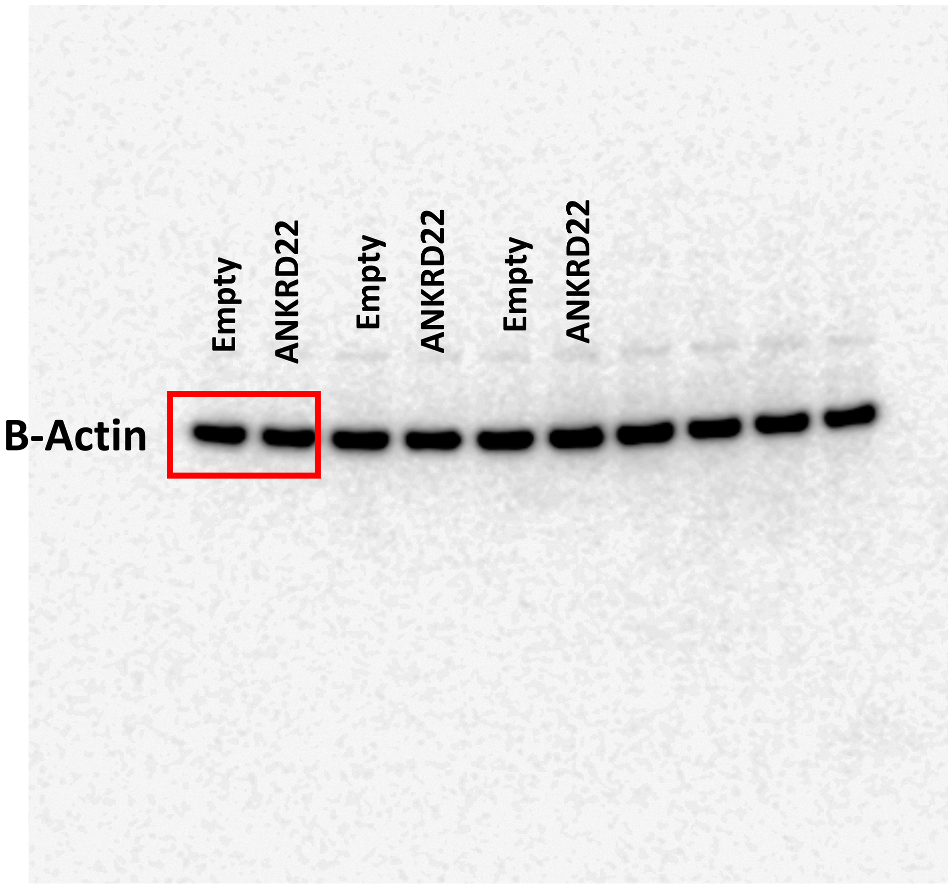


Red box area are used in the figure

Fig 7C.

Red box area are used in the figure

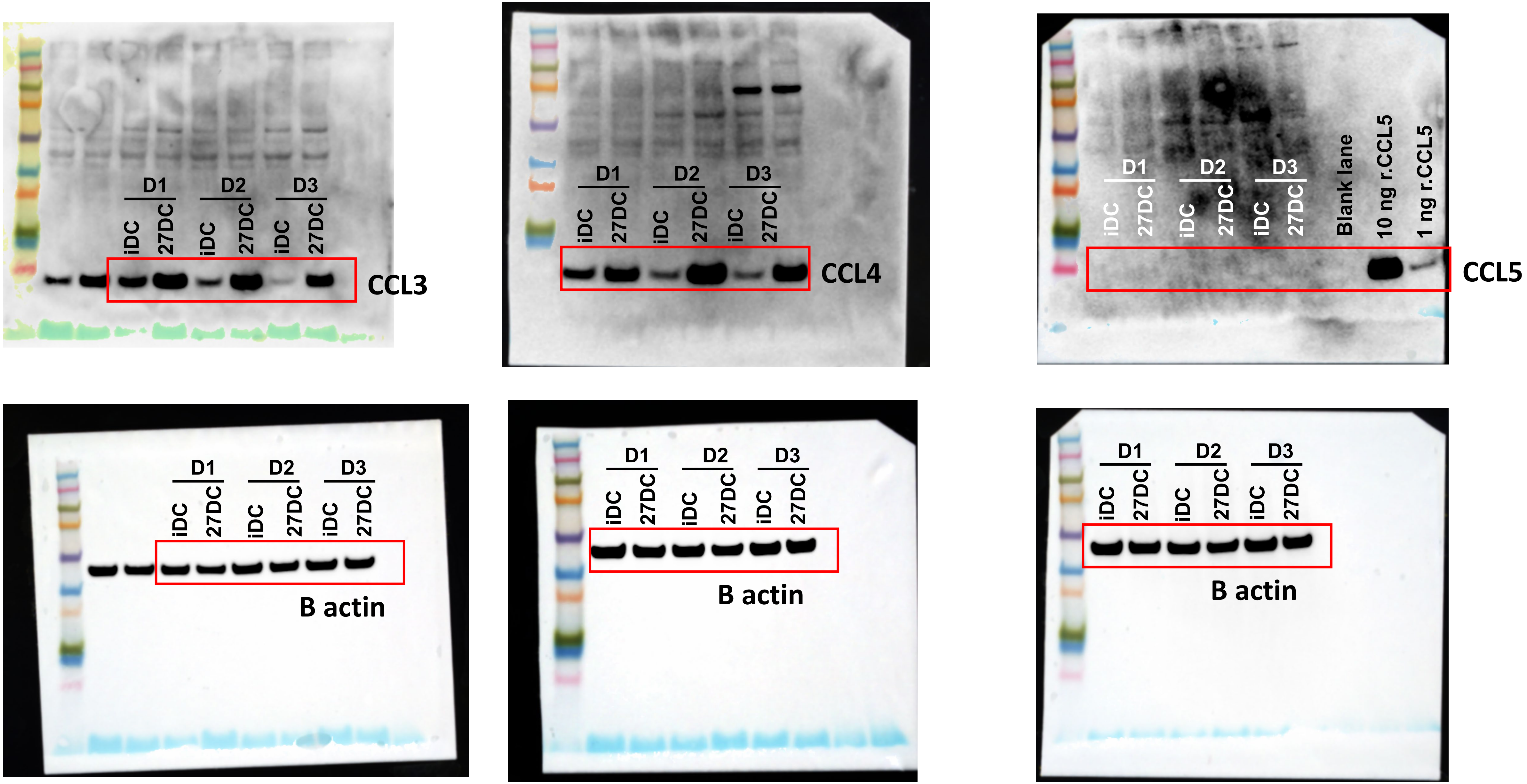

S1 Fig

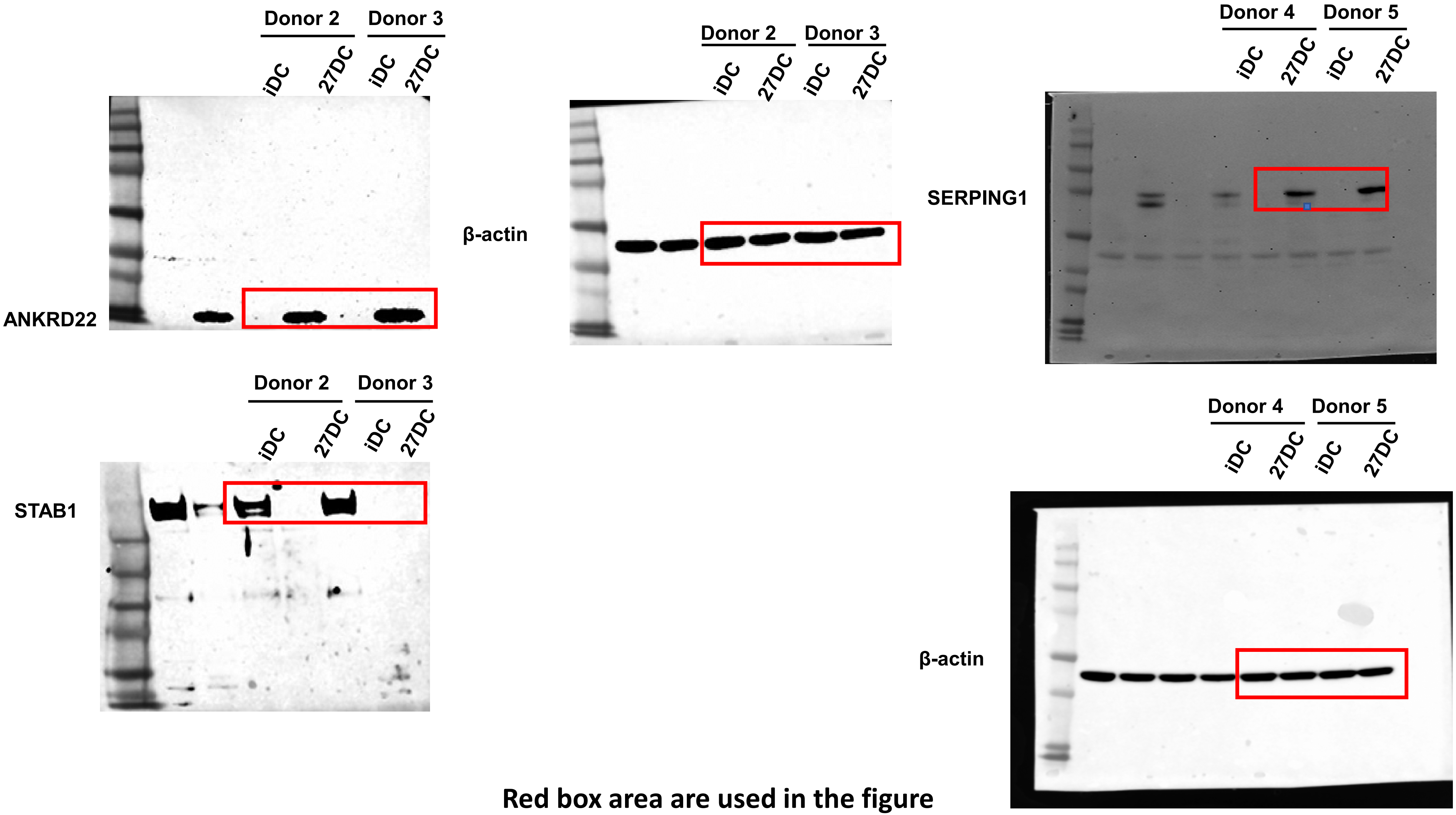


Red box area are used in the figure
