# Supplementary material for: Interleukin-27-induced HIV-resistant dendritic cells suppress reveres transcription following virus entry in an SPTBN1, autophagy, and YB-1 independent manner
Source: PLoS One. 2023 Nov 1;18(11):e0287829. doi: 10.1371/journal.pone.0287829 (PMC10619827; doi:10.1371/journal.pone.0287829)
Supplement: Attachment — Submitted filename: point by point respsonse.docx [file pone.0287829.s006.docx]

**Reviewer #1:** Authors have beautifully shown the mechanism of HIV resistance in 27iDCs. As mentioned by authors, they failed to deduce the molecular details due to limitation of transfection efficiency and cell death. Authors should also discuss the ways they can imagine for understanding the molecular details.

***Responses: As we failed to define the mechanism of anti-HIV effect in 27DC, we currently consider the ant-HIV effect may be mediated by post translational modifications of uncharacterized factor or a change in metabolism(s). The study is under way. Those statement has been incorporated in lines 715-720 in the conclusion section.***

I need authors to revise the manuscript for a few typos.

***Responses: Typos were corrected.***

Additionally, increase font size in Fig 2B and keep same font size in all figures.

***Responses: Fig 2B was revised with increasing font size and kept same font size.***

**Reviewer #2:** In the manuscript by Imamichi et al., the authors investigated the function of IL-27-induced iDC (27DC) on HIV infection. They also observed that the inhibitory mechanism of IL-27-induced HIV resistance in DCs differs from that observed in macrophages and T cells. But currently they do not know the molecular mechanisms underlying the inhibition in 27DC.
Although the data presented in the manuscript is interesting and concrete, further experiments need to be done to strengthen the manuscript.
I have a few major/minor suggestions for polishing the manuscript.

Major comments:

1. Figure 4A, the authors have used two-dimensional western blot to detect the level of acetylation in YB1 protein. The authors should use additional experiments like mass spectrometry to check their results.

**Response: As we mentioned in the Introduction section of the original MS, in our previous work (D. Poudyal. et al., AIDS, 2019, 33:1819-1830. DOI:** [**10.1097/QAD.0000000000002288**](https://doi.org/10.1097/qad.0000000000002288)**), IL-27-treated T cells induced a change in mobility of YB-1 in** **two-dimensional western blot (2DWB) compared to untreated T cells, subsequently the cells resisted HIV infection by inhibiting HIV RT reaction.**

**We demonstrated that the change in the mobility of YB-1 was caused by the decrease in acetylation of YB-1, using mass spirometry analysis. In the current work, we used the same technique to compare the mobility of YB-1 between iDC and 27DC. As shown in the original Fig 4A, we have not observed any significant changes in the mobility between iDC and 27DC, indicating that post-translational modification including the acetylation is not changed; therefore, we did not conduct further analysis of YB-1. To clearly describe the statement, *we have provided the information in Introduction section lines 97-111 and 112 and modified the text in lines 478-479, 486-488, and 490-493.***

2. Figure 4C, the image is a bit blurry, the results look convincing, but they should replace it with a better resolution image.

***Response. Based on the author instruction of the initial submission, we had provided low resolution of images. According to the instruction for the revision, we have now provided higher resolution of images, and the images size of the figure make larger in the revised Fig 4C.***

3. Figure 5C, Is the quantification done with three biological replicates or with one replicate only? The authors should put a separate graph quantifying the three biological replicates from the western blot of ANKRD22, SERPING1 and STAB 1.

***Response.*  *The quantification of Figure 5C in the original manuscript was one replicate to demonstrate the protein expression associated with the gene expression. In response to the reviewer’s comment, we provided two additional Western blotting data in S1 Fig. The quantification data was statistically analyzed, and data is provided in the text in lines 577-578 and 579.***

4. The authors should state why they chose ANKRD22 and SERPING 1 for further analysis in the section ‘Comparison of gene expression profiles.’

***Response. As described in the original MS, we chose the two genes for further analysis after cross reference analysis with the literature research, since ANKRD22 and SERPING1 have not been investigated anti-HIV effect. To precisely describe, we have modified the paragraph in lines 545-568 in the revised MS. (Please see our comment for #8 below).***

5. The authors mentioned in Lines 559-561, ‘Transfection of siRNA induces…...HIV infection’. Did the authors check the levels of IFN1 and IFIII themselves. If not, they should validate this data themselves.

**Response. In the current study, we had not evaluated the IFN induction level after siRNA transfection. As shown in Fig 6B of the original and revised MS, none-target siRNA (si-Ctlr) perse suppressed HIV infection in iDCs. This result indicated that the transfection of siRNA into iDCs induces an off-target effect and then inhibited HIV. We mentioned IFN as one of off-target effect in the original MS, but did not purse the effect as we thought that was out of our current goal.**

**In response to the reviewer’s comment, we assessed whether IFN is induced after siRNA transfection in iDC. Since siRNA transfection suppressed HIV infection in iDC, which is a control, we assessed the IFN inducing effect in iDCs. The si-Ctlr or si-ANKRD22 transfection induced IFNs in iDC, indicating that the IFN activation may suppress the HIV infection in iDC, thus we cannot use siRNA in our assay using iDCs to define a role of host factors. We have now incorporated the result of IFN quantification in S2. Fig, and discussed the result in lines 604-614.**

6. In Figure 7A, C, although the ELISA results were convincing, the authors should conduct western blot to check the levels of CCL3, CCL4, CCL5 and SERPING1.

***Response.  In response to the reviewer’s comment, we conducted WB for detecting intracellular CCL3, CCL4 and CCL5 using whole cell lysate from iDC and 27DC. CCL3 and CCL4 proteins in 27DC was increased, but the expression of CCL5 was not detected. We have incorporated the results in Fig. 7C (The original Fig. 7C is now Fig. 7D) and the results were described in lines 660-661, 668-670, and 671-673, and discussed in line 670. The information for antibodies used for this WB has been provided in the Materials and Methods section in lines 247-248. Figure legend for the new Fig 7C has been provide in lines 682-688. Original Figure 7C legend is now D (ling 688).***

***The result of WB of SERPING1 has been provided in Figure 5C in the original MS and now we have provided additional data from two different donors in Supplemental S1 Fig (please see our response to the major comment #3 above).***

7. In Page 654, the authors claimed that ‘CCL3 protects against R5 HIV infection in DC, macrophages, and T cells; therefore, if 27DC is cultured with other cell types, macrophages or T cells, the secreted CCL3 may inhibit HIV infection in the cocultured cells by trans’. The authors should conduct further experiments to revalidate this hypothesis.

***Response.  The goal of the current study is to define the anti-HIV property of 27DC. The paragraph in page 654 of the original MS was a part of discussion and described perspective. To prove the hypothesis is out of our aim in this current study. In response to the comment, we have incorporated a statement in lines 722-726.***

8. As the authors currently do not know how HIV infection is inhibited in 27DC, they can randomly pick up few targets from the remaining 16 genes. They can check the protein and gene expression of these few targets. Even though the authors gave suitable explanations of why they do not include them in their analysis. Testing few of them can give some novel insights about the mechanisms.

**Response. As described in the original text (lines 389), after HIV infection, HIV core protein is released in the cytosol of the infected cells. The RT reaction processes inside the core protein which protects the RT reaction from the cytosolic envelopment. In 27DC, the RT reaction was inhibited; therefore, we considered the inhibitory factors are cytosolic proteins, but neither cell surface proteins nor the nuclear proteins. As described in the original MS, of the remaining 16 genes, ten genes encode receptors or transcriptional factors, and six have already been characterized as anti-HIV proteins; they decrease infectability or inhibit translation of viral proteins, which are not affected on 27DC. In the current study, we failed to determine the mechanism of the anti-HIV effect in 27DC, since knockdown or induction of the gene of interest cannot be conducted using primary dendritic cells with currently available technologies. We are currently preparing a manuscript focusing on changes in post-translational modification and cell metabolism which affects the RT reaction. To clearly describe we have modified the text in lines 545-568.**

***In response to the editor's request, in the remaining 16 genes, we conducted several assays focusing on GBP4. The reason why we chose GBP4 is that the biological function of the gene product has yet to be clearly described in the remaining 16 genes. As shown below, qRT-PCR (A) and WB (B) confirmed the expression of the gene and gene products, respectively. We assessed the impact of overexpression of GBP4 on HIV infection in HEK293T cells by either single- or co-transfection with ANKRD22. Although the intended gene products were induced (C), none of the expressions had any significant impact on HIV infection (D).***

*Figure Legend:*

*(A) Total cellular RNA of iDC and 27DC from three independent donors were subjected for qRT-PCR using GBP4 and GAPDH probes. Data show mean ± SEM. ***: p<0.001 (B) The total cellular protein lysate from iDC and 27DC were subject for Western blotting using anti-GBP4 (Proteintech, Cat#17746-1-AP: used at 1:500 dilution), anti-ANKRD22 and anti-GAPDH antibodies. (C, D). HEK293T cells were transfected with Empty plasmid (pEmpty), ANKRD22-encoding plasmid (pANKRD22) or GBP4-encoding plasmids (pGBP4) for 48 hours, aliquots of cells were subject for Western blot to detect ANKRD 22 and GBP4 expression (C) and remaining cells were infected with HIV-Luc (D) and then HIV infection was quantified by Luciferase activity. Representative data from two independent assays are presented as means ± SDs (n = 4).*

Minor comments:

1. In the Introduction section write something about HIV infection in one or two sentences before Line 54. Then continue with Line 54.

***Response. We have incorporated a paragraph explaining HIV infection in lines 57-64.***

2. In Line 97-98 the authors say ‘Therefore, in the current study…….27DC.” They should say something more about their current study in the introduction section.

***Response.  In response to the reviewer’s comment, we have incorporated our goal in the current study in lines 118-120.***

3. Line 157, the authors mentioned ‘Passive Lysis Buffer’ please indicate the composition or brand name along with this.

**Response: We obtained the buffer from Promega, we have incorporated the information in the revised MS in lines 179 and 180.**

4. In the Materials and Methods section, the authors should put Quantitative RT-PCR protocol between HIV-1 binding Assay and HIV-1 Fusion Assay.

**Response: We have changed it accordingly in lines 189-196.**

5. Line 202, please omit extra bracket and full stop at the end of the sentence.

**Response: We have corrected it in line 232.**

6. Line 207, the authors should end the sentence in ‘15 minutes on ice’. Then start a new sentence with ‘Then cell debris…’.

**Response: We have modified the sentence accordingly in line 237.**

7. Line 216, Line 222, the authors should mention the dilution of the antibodies used in the experiment.

**Response: We have incorporated dilution factors for each antibody in the Methods section in lines 250-251, 252-253 and 255.**

8. Line 267, change 9700 with 970C.

**Response: The instrument is 9700 (line 197 in the revised MS).**

9. Line 286, change 10*10^6 with 1*10^7.

**Response: We have revised the cell number accordingly in line 320.**

10. In the Materials and Methods Section, the authors should indicate p-values in a more elaborate manner. Like p-values with less than 0.05 as *, less than 0.01 as ** and less than 0.001 as *** and so on. Also, SE should be replaced with SEM.

**Response: We have incorporated the p-value with the elaborating manners as the reviewer suggested in lines 345-346 and all SE was replaced with SEM (lines 343, 369, 377, 407, 446, 511, 535, 682, 691), accordingly.**

11. Line 322, please indicate the exact p-values.

**Response: We recognized that in the original text, we had a typo in the p-value for HIVNL4.3; it should have been shown p<0.05 as shown in the original figure. We have now provided the extract p values in the text in response to the reviewer's comment in lines 357.**

12. Line 323, the authors say that ‘As the replication activity of the X4 virus is low in iDC…...’ Although it is previously reported, the authors can add a line explaining what is happening here as per previous reports.

**Response: In response to the comment, we have incorporated our previous work to explain why X4 HIV replicates less than R5 virus in lines 359-360.**

13. In Figure 1A and 1B, the authors can add which one is HIVAD8 and HIVNL4.3 in the figure itself. Although it is added in the Figure legend, it will be more helpful if it is in the figure itself.

***Response: We have inserted HIVAD8 and HIVNL4.3 in the figures.***

14. In many instances the authors say means ± SE/SD, it should be changed to Mean± SEM/SD.

***Response: We have changed all SE to SEM (please see our response to the minor comment #10 above).***

15. In Figure 3C and 7C, the authors indicate ****, what will be its corresponding p-value. The authors did not mention anything about it in Line 420 and Line 627 respectively.

***Response.  We have provided the meaning of **** in the text in lines 454 and 691. The authors appreciate the comment.***

16. Line 364, 423, 443, 488 mention the exact corresponding p-value.

**Response: We have inserted the exact p- value in the lines 400, 457, and 477. The p<0.05 in line 488 of the original MS (line 542 in the revised MS) described summary of p-values, in response to the reviewer’s comment, all p-values for the selected genes have been incorporated in the revised S1 Table.**

17. Figure 3B, during comparison of iDC+ TAK779 and 27DC + TAK779 put ns.

***Response: We have incorporated n.s. in the figure.***

18. Figure 4D, the y-axis should be set to 0.5, this will make the figure good.

**Response: As shown below, if the Y-axis is set to 0.5, it is difficult to see the value of iDC (the top figure below); if it is set to 0.5, it can read the value (bottom figure below); thus, we changed the set to 0.05, and the revised figure is now provided as Figure 4D. I wish it would be acceptable.**

19. Page 25, Line 547, do not mention which reagents are used for the experiment. Discuss this in the Materials and Methods section.

***Response: We have modified the text accordingly in lines 306-314.***

20. Line 604, indicate the corresponding p-value after 1.37±0.28 fold (n=4).

***Response: We have incorporated the value in 659 in the revised MS.***

21. Line 635, the authors should put a space between 27DC and protein levels.

***Response: We have revised the figure accordingly in Line 699 in the revised version.***

22. Figure 7B, the authors should include iDC and 27DC below black and grey bars respectively.

***Response: We have revised the figure accordingly.***

23. Line 659, check the spelling of SERPING.

***Response: We have corrected accordingly in Line 730 in the revised version.***
