# Supplementary material for: Interleukin-27-induced HIV-resistant dendritic cells suppress reveres transcription following virus entry in an SPTBN1, autophagy, and YB-1 independent manner
Source: PLoS One. 2023 Nov 1;18(11):e0287829. doi: 10.1371/journal.pone.0287829 (PMC10619827; doi:10.1371/journal.pone.0287829)
Supplement: Peer review history

# Peer review: Decision Letter 0

**DOI:** 10.1371/journal.pone.0287829.r001

**Authors:** Arunava Roy

15 Aug 2023

PONE-D-23-18217Interleukin-27-induced HIV-resistant dendritic cells suppress reveres transcription following virus entry in an SPTBN1, Autophagy, and YB-1 independent mannerPLOS ONE

Dear Dr. Imamichi,

Thank you for submitting your manuscript to PLOS ONE. After careful consideration, we feel that it has merit but does not fully meet PLOS ONE’s publication criteria as it currently stands. Therefore, we invite you to submit a revised version of the manuscript that addresses the points raised during the review process.

Please submit your revised manuscript by Sep 29 2023 11:59PM. If you will need more time than this to complete your revisions, please reply to this message or contact the journal office at plosone@plos.org. When you're ready to submit your revision, log on to https://www.editorialmanager.com/pone/ and select the 'Submissions Needing Revision' folder to locate your manuscript file.

Please include the following items when submitting your revised manuscript:

- A rebuttal letter that responds to each point raised by the academic editor and reviewer(s). You should upload this letter as a separate file labeled 'Response to Reviewers'.
- A marked-up copy of your manuscript that highlights changes made to the original version. You should upload this as a separate file labeled 'Revised Manuscript with Track Changes'.
- An unmarked version of your revised paper without tracked changes. You should upload this as a separate file labeled 'Manuscript'.

If you would like to make changes to your financial disclosure, please include your updated statement in your cover letter. Guidelines for resubmitting your figure files are available below the reviewer comments at the end of this letter.

If applicable, we recommend that you deposit your laboratory protocols in protocols.io to enhance the reproducibility of your results. Protocols.io assigns your protocol its own identifier (DOI) so that it can be cited independently in the future. For instructions see: https://journals.plos.org/plosone/s/submission-guidelines#loc-laboratory-protocols. Additionally, PLOS ONE offers an option for publishing peer-reviewed Lab Protocol articles, which describe protocols hosted on protocols.io. Read more information on sharing protocols at https://plos.org/protocols?utm_medium=editorial-email&utm_source=authorletters&utm_campaign=protocols.

We look forward to receiving your revised manuscript.

Kind regards,

Arunava Roy, Ph.D.

Academic Editor

PLOS ONE

Journal Requirements:

When submitting your revision, we need you to address these additional requirements.

1. Please ensure that your manuscript meets PLOS ONE's style requirements, including those for file naming. The PLOS ONE style templates can be found at

https://journals.plos.org/plosone/s/file?id=wjVg/PLOSOne_formatting_sample_main_body.pdf and

https://journals.plos.org/plosone/s/file?id=ba62/PLOSOne_formatting_sample_title_authors_affiliations.pdf.

2. . PLOS requires an ORCID iD for the corresponding author in Editorial Manager on papers submitted after December 6th, 2016. Please ensure that you have an ORCID iD and that it is validated in Editorial Manager. To do this, go to ‘Update my Information’ (in the upper left-hand corner of the main menu), and click on the Fetch/Validate link next to the ORCID field. This will take you to the ORCID site and allow you to create a new iD or authenticate a pre-existing iD in Editorial Manager. Please see the following video for instructions on linking an ORCID iD to your Editorial Manager account: https://www.youtube.com/watch?v=_xcclfuvtxQ.

3. Thank you for stating the following in the Acknowledgments Section of your manuscript:

“This project has been funded in whole or in part with federal funds from the National Cancer Institute, National Institutes of Health, under Contract No. HHSN261200800001E.”

We note that you have provided additional information within the Acknowledgements Section that is not currently declared in your Funding Statement. Please note that funding information should not appear in the Acknowledgments section or other areas of your manuscript. We will only publish funding information present in the Funding Statement section of the online submission form.

Please remove any funding-related text from the manuscript and let us know how you would like to update your Funding Statement. Currently, your Funding Statement reads as follows:

“This project has been funded in whole or in part with federal funds from the National Cancer Institute, National Institutes of Health, under Contract No. HHSN261200800001E.”

Please include your amended statements within your cover letter; we will change the online submission form on your behalf.

4. We note that you have included the phrase “data not shown” in your manuscript. Unfortunately, this does not meet our data sharing requirements. PLOS does not permit references to inaccessible data. We require that authors provide all relevant data within the paper, Supporting Information files, or in an acceptable, public repository. Please add a citation to support this phrase or upload the data that corresponds with these findings to a stable repository (such as Figshare or Dryad) and provide and URLs, DOIs, or accession numbers that may be used to access these data. Or, if the data are not a core part of the research being presented in your study, we ask that you remove the phrase that refers to these data.

5. PLOS ONE now requires that authors provide the original uncropped and unadjusted images underlying all blot or gel results reported in a submission’s figures or Supporting Information files. This policy and the journal’s other requirements for blot/gel reporting and figure preparation are described in detail at https://journals.plos.org/plosone/s/figures#loc-blot-and-gel-reporting-requirements and https://journals.plos.org/plosone/s/figures#loc-preparing-figures-from-image-files. When you submit your revised manuscript, please ensure that your figures adhere fully to these guidelines and provide the original underlying images for all blot or gel data reported in your submission. See the following link for instructions on providing the original image data: https://journals.plos.org/plosone/s/figures#loc-original-images-for-blots-and-gels.

In your cover letter, please note whether your blot/gel image data are in Supporting Information or posted at a public data repository, provide the repository URL if relevant, and provide specific details as to which raw blot/gel images, if any, are not available. Email us at plosone@plos.org if you have any questions."

6. Please include captions for your Supporting Information files at the end of your manuscript, and update any in-text citations to match accordingly. Please see our Supporting Information guidelines for more information: http://journals.plos.org/plosone/s/supporting-information.

[Note: HTML markup is below. Please do not edit.]

Reviewers' comments:

Reviewer's Responses to Questions

**Comments to the Author**

1. Is the manuscript technically sound, and do the data support the conclusions?

The manuscript must describe a technically sound piece of scientific research with data that supports the conclusions. Experiments must have been conducted rigorously, with appropriate controls, replication, and sample sizes. The conclusions must be drawn appropriately based on the data presented. 

Reviewer #1: Yes

Reviewer #2: Yes

**********

2. Has the statistical analysis been performed appropriately and rigorously? 

Reviewer #1: Yes

Reviewer #2: Yes

**********

3. Have the authors made all data underlying the findings in their manuscript fully available?

The PLOS Data policy requires authors to make all data underlying the findings described in their manuscript fully available without restriction, with rare exception (please refer to the Data Availability Statement in the manuscript PDF file). The data should be provided as part of the manuscript or its supporting information, or deposited to a public repository. For example, in addition to summary statistics, the data points behind means, medians and variance measures should be available. If there are restrictions on publicly sharing data—e.g. participant privacy or use of data from a third party—those must be specified.

Reviewer #1: Yes

Reviewer #2: Yes

**********

4. Is the manuscript presented in an intelligible fashion and written in standard English?

PLOS ONE does not copyedit accepted manuscripts, so the language in submitted articles must be clear, correct, and unambiguous. Any typographical or grammatical errors should be corrected at revision, so please note any specific errors here.

Reviewer #1: Yes

Reviewer #2: Yes

**********

5. Review Comments to the Author

Please use the space provided to explain your answers to the questions above. You may also include additional comments for the author, including concerns about dual publication, research ethics, or publication ethics. (Please upload your review as an attachment if it exceeds 20,000 characters)

Reviewer #1: Authors have beautifully shown the mechanism of HIV resistance in 27iDCs. As mentioned by authors, they failed to deduce the molecular details due to limitation of transfection efficiency and cell death. Authors should also discuss the ways they can imagine for understanding the molecular details. I need authors to revise the manuscript for a few typos. Additionally, increase font size in Fig 2B and keep same font size in all figures.

Reviewer #2: In the manuscript by Imamichi et al., the authors investigated the function of IL-27-induced iDC (27DC) on HIV infection. They also observed that the inhibitory mechanism of IL-27-induced HIV resistance in DCs differs from that observed in macrophages and T cells. But currently they do not know the molecular mechanisms underlying the inhibition in 27DC.

Although the data presented in the manuscript is interesting and concrete, further experiments need to be done to strengthen the manuscript.

I have a few major/minor suggestions for polishing the manuscript.

Major comments:

1. Figure 4A, the authors have used two-dimensional western blot to detect the level of acetylation in YB1 protein. The authors should use additional experiments like mass spectrometry to check their results.

2. Figure 4C, the image is a bit blurry, the results look convincing, but they should replace it with a better resolution image.

3. Figure 5C, Is the quantification done with three biological replicates or with one replicate only? The authors should put a separate graph quantifying the three biological replicates from the western blot of ANKRD22, SERPING1 and STAB 1.

4. The authors should state why they chose ANKRD22 and SERPING 1 for further analysis in the section ‘Comparison of gene expression profiles.’

5. The authors mentioned in Lines 559-561, ‘Transfection of siRNA induces…...HIV infection’. Did the authors check the levels of IFN1 and IFIII themselves. If not, they should validate this data themselves.

6. In Figure 7A, C, although the ELISA results were convincing, the authors should conduct western blot to check the levels of CCL3, CCL4, CCL5 and SERPING1.

7. In Page 654, the authors claimed that ‘CCL3 protects against R5 HIV infection in DC, macrophages, and T cells; therefore, if 27DC is cultured with other cell types, macrophages or T cells, the secreted CCL3 may inhibit HIV infection in the cocultured cells by trans’. The authors should conduct further experiments to revalidate this hypothesis.

8. As the authors currently do not know how HIV infection is inhibited in 27DC, they can randomly pick up few targets from the remaining 16 genes. They can check the protein and gene expression of these few targets. Even though the authors gave suitable explanations of why they do not include them in their analysis. Testing few of them can give some novel insights about the mechanisms.

Minor comments:

1. In the Introduction section write something about HIV infection in one or two sentences before Line 54. Then continue with Line 54.

2. In Line 97-98 the authors say ‘Therefore, in the current study…….27DC.” They should say something more about their current study in the introduction section.

3. Line 157, the authors mentioned ‘Passive Lysis Buffer’ please indicate the composition or brand name along with this.

4. In the Materials and Methods section, the authors should put Quantitative RT-PCR protocol between HIV-1 binding Assay and HIV-1 Fusion Assay.

5. Line 202, please omit extra bracket and full stop at the end of the sentence.

6. Line 207, the authors should end the sentence in ‘15 minutes on ice’. Then start a new sentence with ‘Then cell debris…’.

7. Line 216, Line 222, the authors should mention the dilution of the antibodies used in the experiment.

8. Line 267, change 9700 with 970C.

9. Line 286, change 10*10^6 with 1*10^7.

10. In the Materials and Methods Section, the authors should indicate p-values in a more elaborate manner. Like p-values with less than 0.05 as *, less than 0.01 as ** and less than 0.001 as *** and so on. Also, SE should be replaced with SEM.

11. Line 322, please indicate the exact p-values.

12. Line 323, the authors say that ‘As the replication activity of the X4 virus is low in iDC…...’ Although it is previously reported, the authors can add a line explaining what is happening here as per previous reports.

13. In Figure 1A and 1B, the authors can add which one is HIVAD8 and HIVNL4.3 in the figure itself. Although it is added in the Figure legend, it will be more helpful if it is in the figure itself.

14. In many instances the authors say means ± SE/SD, it should be changed to Mean± SEM/SD.

15. In Figure 3C and 7C, the authors indicate ****, what will be its corresponding p-value. The authors did not mention anything about it in Line 420 and Line 627 respectively.

16. Line 364, 423, 443, 488 mention the exact corresponding p-value.

17. Figure 3B, during comparison of iDC+ TAK779 and 27DC + TAK779 put ns.

18. Figure 4D, the y-axis should be set to 0.5, this will make the figure good.

19. Page 25, Line 547, do not mention which reagents are used for the experiment. Discuss this in the Materials and Methods section.

20. Line 604, indicate the corresponding p-value after 1.37±0.28 fold (n=4).

21. Line 635, the authors should put a space between 27DC and protein levels.

22. Figure 7B, the authors should include iDC and 27DC below black and grey bars respectively.

23. Line 659, check the spelling of SERPING.

**********

6. PLOS authors have the option to publish the peer review history of their article (what does this mean?). If published, this will include your full peer review and any attached files.

If you choose “no”, your identity will remain anonymous but your review may still be made public.

**Do you want your identity to be public for this peer review?** For information about this choice, including consent withdrawal, please see our Privacy Policy.

Reviewer #1: **Yes: **Shilpi Giri

Reviewer #2: No

**********

[NOTE: If reviewer comments were submitted as an attachment file, they will be attached to this email and accessible via the submission site. Please log into your account, locate the manuscript record, and check for the action link "View Attachments". If this link does not appear, there are no attachment files.]

While revising your submission, please upload your figure files to the Preflight Analysis and Conversion Engine (PACE) digital diagnostic tool, https://pacev2.apexcovantage.com/. PACE helps ensure that figures meet PLOS requirements. To use PACE, you must first register as a user. Registration is free. Then, login and navigate to the UPLOAD tab, where you will find detailed instructions on how to use the tool. If you encounter any issues or have any questions when using PACE, please email PLOS at figures@plos.org. Please note that Supporting Information files do not need this step.

# Author comment: Author response to Decision Letter 0

**DOI:** 10.1371/journal.pone.0287829.r002

11 Sep 2023

Reviewer #1: Authors have beautifully shown the mechanism of HIV resistance in 27iDCs. As mentioned by authors, they failed to deduce the molecular details due to limitation of transfection efficiency and cell death. Authors should also discuss the ways they can imagine for understanding the molecular details. 

Responses: As we failed to define the mechanism of anti-HIV effect in 27DC, we currently consider the ant-HIV effect may be mediated by post translational modifications of uncharacterized factor or a change in metabolism(s). The study is under way. Those statement has been incorporated in lines 715-720 in the conclusion section.

I need authors to revise the manuscript for a few typos. 

Responses: Typos were corrected.

Additionally, increase font size in Fig 2B and keep same font size in all figures.

Responses: Fig 2B was revised with increasing font size and kept same font size.

Reviewer #2: In the manuscript by Imamichi et al., the authors investigated the function of IL-27-induced iDC (27DC) on HIV infection. They also observed that the inhibitory mechanism of IL-27-induced HIV resistance in DCs differs from that observed in macrophages and T cells. But currently they do not know the molecular mechanisms underlying the inhibition in 27DC.

Although the data presented in the manuscript is interesting and concrete, further experiments need to be done to strengthen the manuscript.

I have a few major/minor suggestions for polishing the manuscript.

Major comments:

1. Figure 4A, the authors have used two-dimensional western blot to detect the level of acetylation in YB1 protein. The authors should use additional experiments like mass spectrometry to check their results.

Response: As we mentioned in the Introduction section of the original MS, in our previous work (D. Poudyal. et al., AIDS, 2019, 33:1819-1830. DOI: 10.1097/QAD.0000000000002288), IL-27-treated T cells induced a change in mobility of YB-1 in two-dimensional western blot (2DWB) compared to untreated T cells, subsequently the cells resisted HIV infection by inhibiting HIV RT reaction. 

We demonstrated that the change in the mobility of YB-1 was caused by the decrease in acetylation of YB-1, using mass spirometry analysis. In the current work, we used the same technique to compare the mobility of YB-1 between iDC and 27DC. As shown in the original Fig 4A, we have not observed any significant changes in the mobility between iDC and 27DC, indicating that post-translational modification including the acetylation is not changed; therefore, we did not conduct further analysis of YB-1. To clearly describe the statement, we have provided the information in Introduction section lines 97-111 and 112 and modified the text in lines 478-479, 486-488, and 490-493. 

2. Figure 4C, the image is a bit blurry, the results look convincing, but they should replace it with a better resolution image.

Response. Based on the author instruction of the initial submission, we had provided low resolution of images. According to the instruction for the revision, we have now provided higher resolution of images, and the images size of the figure make larger in the revised Fig 4C.

3. Figure 5C, Is the quantification done with three biological replicates or with one replicate only? The authors should put a separate graph quantifying the three biological replicates from the western blot of ANKRD22, SERPING1 and STAB 1.

Response. The quantification of Figure 5C in the original manuscript was one replicate to demonstrate the protein expression associated with the gene expression. In response to the reviewer’s comment, we provided two additional Western blotting data in S1 Fig. The quantification data was statistically analyzed, and data is provided in the text in lines 577-578 and 579.

4. The authors should state why they chose ANKRD22 and SERPING 1 for further analysis in the section ‘Comparison of gene expression profiles.’

Response. As described in the original MS, we chose the two genes for further analysis after cross reference analysis with the literature research, since ANKRD22 and SERPING1 have not been investigated anti-HIV effect. To precisely describe, we have modified the paragraph in lines 545-568 in the revised MS. (Please see our comment for #8 below).

5. The authors mentioned in Lines 559-561, ‘Transfection of siRNA induces…...HIV infection’. Did the authors check the levels of IFN1 and IFIII themselves. If not, they should validate this data themselves.

Response. In the current study, we had not evaluated the IFN induction level after siRNA transfection. As shown in Fig 6B of the original and revised MS, none-target siRNA (si-Ctlr) perse suppressed HIV infection in iDCs. This result indicated that the transfection of siRNA into iDCs induces an off-target effect and then inhibited HIV. We mentioned IFN as one of off-target effect in the original MS, but did not purse the effect as we thought that was out of our current goal. 

In response to the reviewer’s comment, we assessed whether IFN is induced after siRNA transfection in iDC. Since siRNA transfection suppressed HIV infection in iDC, which is a control, we assessed the IFN inducing effect in iDCs. The si-Ctlr or si-ANKRD22 transfection induced IFNs in iDC, indicating that the IFN activation may suppress the HIV infection in iDC, thus we cannot use siRNA in our assay using iDCs to define a role of host factors. We have now incorporated the result of IFN quantification in S2. Fig, and discussed the result in lines 604-614.

6. In Figure 7A, C, although the ELISA results were convincing, the authors should conduct western blot to check the levels of CCL3, CCL4, CCL5 and SERPING1.

Response. In response to the reviewer’s comment, we conducted WB for detecting intracellular CCL3, CCL4 and CCL5 using whole cell lysate from iDC and 27DC. CCL3 and CCL4 proteins in 27DC was increased, but the expression of CCL5 was not detected. We have incorporated the results in Fig. 7C (The original Fig. 7C is now Fig. 7D) and the results were described in lines 660-661, 668-670, and 671-673, and discussed in line 670. The information for antibodies used for this WB has been provided in the Materials and Methods section in lines 247-248. Figure legend for the new Fig 7C has been provide in lines 682-688. Original Figure 7C legend is now D (ling 688).

 The result of WB of SERPING1 has been provided in Figure 5C in the original MS and now we have provided additional data from two different donors in Supplemental S1 Fig (please see our response to the major comment #3 above).

7. In Page 654, the authors claimed that ‘CCL3 protects against R5 HIV infection in DC, macrophages, and T cells; therefore, if 27DC is cultured with other cell types, macrophages or T cells, the secreted CCL3 may inhibit HIV infection in the cocultured cells by trans’. The authors should conduct further experiments to revalidate this hypothesis.

Response. The goal of the current study is to define the anti-HIV property of 27DC. The paragraph in page 654 of the original MS was a part of discussion and described perspective. To prove the hypothesis is out of our aim in this current study. In response to the comment, we have incorporated a statement in lines 722-726. 

8. As the authors currently do not know how HIV infection is inhibited in 27DC, they can randomly pick up few targets from the remaining 16 genes. They can check the protein and gene expression of these few targets. Even though the authors gave suitable explanations of why they do not include them in their analysis. Testing few of them can give some novel insights about the mechanisms.

Response. As described in the original text (lines 389), after HIV infection, HIV core protein is released in the cytosol of the infected cells. The RT reaction processes inside the core protein which protects the RT reaction from the cytosolic envelopment. In 27DC, the RT reaction was inhibited; therefore, we considered the inhibitory factors are cytosolic proteins, but neither cell surface proteins nor the nuclear proteins. As described in the original MS, of the remaining 16 genes, ten genes encode receptors or transcriptional factors, and six have already been characterized as anti-HIV proteins; they decrease infectability or inhibit translation of viral proteins, which are not affected on 27DC. In the current study, we failed to determine the mechanism of the anti-HIV effect in 27DC, since knockdown or induction of the gene of interest cannot be conducted using primary dendritic cells with currently available technologies. We are currently preparing a manuscript focusing on changes in post-translational modification and cell metabolism which affects the RT reaction. To clearly describe we have modified the text in lines 545-568.

In response to the editor's request, in the remaining 16 genes, we conducted several assays focusing on GBP4. The reason why we chose GBP4 is that the biological function of the gene product has yet to be clearly described in the remaining 16 genes. As shown below, qRT-PCR (A) and WB (B) confirmed the expression of the gene and gene products, respectively. We assessed the impact of overexpression of GBP4 on HIV infection in HEK293T cells by either single- or co-transfection with ANKRD22. Although the intended gene products were induced (C), none of the expressions had any significant impact on HIV infection (D).

Figure Legend:

(A) Total cellular RNA of iDC and 27DC from three independent donors were subjected for qRT-PCR using GBP4 and GAPDH probes. Data show mean ± SEM. ***: p<0.001 (B) The total cellular protein lysate from iDC and 27DC were subject for Western blotting using anti-GBP4 (Proteintech, Cat#17746-1-AP: used at 1:500 dilution), anti-ANKRD22 and anti-GAPDH antibodies. (C, D). HEK293T cells were transfected with Empty plasmid (pEmpty), ANKRD22-encoding plasmid (pANKRD22) or GBP4-encoding plasmids (pGBP4) for 48 hours, aliquots of cells were subject for Western blot to detect ANKRD 22 and GBP4 expression (C) and remaining cells were infected with HIV-Luc (D) and then HIV infection was quantified by Luciferase activity. Representative data from two independent assays are presented as means ± SDs (n = 4).

Minor comments:

1. In the Introduction section write something about HIV infection in one or two sentences before Line 54. Then continue with Line 54.

Response. We have incorporated a paragraph explaining HIV infection in lines 57-64.

2. In Line 97-98 the authors say ‘Therefore, in the current study…….27DC.” They should say something more about their current study in the introduction section.

Response. In response to the reviewer’s comment, we have incorporated our goal in the current study in lines 118-120.

3. Line 157, the authors mentioned ‘Passive Lysis Buffer’ please indicate the composition or brand name along with this.

Response: We obtained the buffer from Promega, we have incorporated the information in the revised MS in lines 179 and 180.

4. In the Materials and Methods section, the authors should put Quantitative RT-PCR protocol between HIV-1 binding Assay and HIV-1 Fusion Assay.

Response: We have changed it accordingly in lines 189-196.

5. Line 202, please omit extra bracket and full stop at the end of the sentence.

Response: We have corrected it in line 232.

6. Line 207, the authors should end the sentence in ‘15 minutes on ice’. Then start a new sentence with ‘Then cell debris…’.

Response: We have modified the sentence accordingly in line 237. 

7. Line 216, Line 222, the authors should mention the dilution of the antibodies used in the experiment.

Response: We have incorporated dilution factors for each antibody in the Methods section in lines 250-251, 252-253 and 255.

8. Line 267, change 9700 with 970C.

Response: The instrument is 9700 (line 197 in the revised MS).

9. Line 286, change 10*10^6 with 1*10^7.

Response: We have revised the cell number accordingly in line 320.

10. In the Materials and Methods Section, the authors should indicate p-values in a more elaborate manner. Like p-values with less than 0.05 as *, less than 0.01 as ** and less than 0.001 as *** and so on. Also, SE should be replaced with SEM.

Response: We have incorporated the p-value with the elaborating manners as the reviewer suggested in lines 345-346 and all SE was replaced with SEM (lines 343, 369, 377, 407, 446, 511, 535, 682, 691), accordingly.

11. Line 322, please indicate the exact p-values.

Response: We recognized that in the original text, we had a typo in the p-value for HIVNL4.3; it should have been shown p<0.05 as shown in the original figure. We have now provided the extract p values in the text in response to the reviewer's comment in lines 357.

12. Line 323, the authors say that ‘As the replication activity of the X4 virus is low in iDC…...’ Although it is previously reported, the authors can add a line explaining what is happening here as per previous reports.

Response: In response to the comment, we have incorporated our previous work to explain why X4 HIV replicates less than R5 virus in lines 359-360.

13. In Figure 1A and 1B, the authors can add which one is HIVAD8 and HIVNL4.3 in the figure itself. Although it is added in the Figure legend, it will be more helpful if it is in the figure itself.

Response: We have inserted HIVAD8 and HIVNL4.3 in the figures.

14. In many instances the authors say means ± SE/SD, it should be changed to Mean± SEM/SD.

Response: We have changed all SE to SEM (please see our response to the minor comment #10 above).

15. In Figure 3C and 7C, the authors indicate ****, what will be its corresponding p-value. The authors did not mention anything about it in Line 420 and Line 627 respectively.

Response. We have provided the meaning of **** in the text in lines 454 and 691. The authors appreciate the comment.

16. Line 364, 423, 443, 488 mention the exact corresponding p-value.

Response: We have inserted the exact p- value in the lines 400, 457, and 477. The p<0.05 in line 488 of the original MS (line 542 in the revised MS) described summary of p-values, in response to the reviewer’s comment, all p-values for the selected genes have been incorporated in the revised S1 Table.

17. Figure 3B, during comparison of iDC+ TAK779 and 27DC + TAK779 put ns.

Response: We have incorporated n.s. in the figure.

18. Figure 4D, the y-axis should be set to 0.5, this will make the figure good.

Response: As shown below, if the Y-axis is set to 0.5, it is difficult to see the value of iDC (the top figure below); if it is set to 0.5, it can read the value (bottom figure below); thus, we changed the set to 0.05, and the revised figure is now provided as Figure 4D. I wish it would be acceptable. 

19. Page 25, Line 547, do not mention which reagents are used for the experiment. Discuss this in the Materials and Methods section.

Response: We have modified the text accordingly in lines 306-314.

20. Line 604, indicate the corresponding p-value after 1.37±0.28 fold (n=4).

Response: We have incorporated the value in 659 in the revised MS.

21. Line 635, the authors should put a space between 27DC and protein levels.

Response: We have revised the figure accordingly in Line 699 in the revised version.

22. Figure 7B, the authors should include iDC and 27DC below black and grey bars respectively.

Response: We have revised the figure accordingly. 

23. Line 659, check the spelling of SERPING. 

Response: We have corrected accordingly in Line 730 in the revised version.

Attachment Submitted filename: point by point respsonse.docx Click here for additional data file.

# Peer review: Decision Letter 1

**DOI:** 10.1371/journal.pone.0287829.r003

**Authors:** Arunava Roy

4 Oct 2023

Interleukin-27-induced HIV-resistant dendritic cells suppress reveres transcription following virus entry in an SPTBN1, Autophagy, and YB-1 independent manner

PONE-D-23-18217R1

Dear Dr. Imamichi,

We’re pleased to inform you that your manuscript has been judged scientifically suitable for publication and will be formally accepted for publication once it meets all outstanding technical requirements.

Within one week, you’ll receive an e-mail detailing the required amendments. When these have been addressed, you’ll receive a formal acceptance letter and your manuscript will be scheduled for publication.

An invoice for payment will follow shortly after the formal acceptance. To ensure an efficient process, please log into Editorial Manager at http://www.editorialmanager.com/pone/, click the 'Update My Information' link at the top of the page, and double check that your user information is up-to-date. If you have any billing related questions, please contact our Author Billing department directly at authorbilling@plos.org.

If your institution or institutions have a press office, please notify them about your upcoming paper to help maximize its impact. If they’ll be preparing press materials, please inform our press team as soon as possible -- no later than 48 hours after receiving the formal acceptance. Your manuscript will remain under strict press embargo until 2 pm Eastern Time on the date of publication. For more information, please contact onepress@plos.org.

Kind regards,

Arunava Roy, Ph.D.

Academic Editor

PLOS ONE

Additional Editor Comments (optional):

Reviewers' comments:

Reviewer's Responses to Questions

**Comments to the Author**

1. If the authors have adequately addressed your comments raised in a previous round of review and you feel that this manuscript is now acceptable for publication, you may indicate that here to bypass the “Comments to the Author” section, enter your conflict of interest statement in the “Confidential to Editor” section, and submit your "Accept" recommendation.

Reviewer #2: All comments have been addressed

**********

2. Is the manuscript technically sound, and do the data support the conclusions?

The manuscript must describe a technically sound piece of scientific research with data that supports the conclusions. Experiments must have been conducted rigorously, with appropriate controls, replication, and sample sizes. The conclusions must be drawn appropriately based on the data presented. 

Reviewer #2: Yes

**********

3. Has the statistical analysis been performed appropriately and rigorously? 

Reviewer #2: Yes

**********

4. Have the authors made all data underlying the findings in their manuscript fully available?

The PLOS Data policy requires authors to make all data underlying the findings described in their manuscript fully available without restriction, with rare exception (please refer to the Data Availability Statement in the manuscript PDF file). The data should be provided as part of the manuscript or its supporting information, or deposited to a public repository. For example, in addition to summary statistics, the data points behind means, medians and variance measures should be available. If there are restrictions on publicly sharing data—e.g. participant privacy or use of data from a third party—those must be specified.

Reviewer #2: Yes

**********

5. Is the manuscript presented in an intelligible fashion and written in standard English?

PLOS ONE does not copyedit accepted manuscripts, so the language in submitted articles must be clear, correct, and unambiguous. Any typographical or grammatical errors should be corrected at revision, so please note any specific errors here.

Reviewer #2: Yes

**********

6. Review Comments to the Author

Please use the space provided to explain your answers to the questions above. You may also include additional comments for the author, including concerns about dual publication, research ethics, or publication ethics. (Please upload your review as an attachment if it exceeds 20,000 characters)

Reviewer #2: In the manuscript by Imamichi et al., the authors investigated the function of IL-27-induced iDC (27DC) on HIV infection. They also observed that the inhibitory mechanism of IL-27-induced HIV resistance in DCs differs from that observed in macrophages and T cells. But currently they do not know the molecular mechanisms underlying the inhibition in 27DC.

The authors have addressed all the comments. The manuscript can be accepted in its present form.

**********

7. PLOS authors have the option to publish the peer review history of their article (what does this mean?). If published, this will include your full peer review and any attached files.

If you choose “no”, your identity will remain anonymous but your review may still be made public.

**Do you want your identity to be public for this peer review?** For information about this choice, including consent withdrawal, please see our Privacy Policy.

Reviewer #2: No

**********

# Editor report: Acceptance letter

**DOI:** 10.1371/journal.pone.0287829.r004

**Authors:** Arunava Roy

24 Oct 2023

PONE-D-23-18217R1 

Interleukin-27-induced HIV-resistant dendritic cells suppress reveres transcription following virus entry in an SPTBN1, autophagy, and YB-1 independent manner. 

Dear Dr. Imamichi:

I'm pleased to inform you that your manuscript has been deemed suitable for publication in PLOS ONE. Congratulations! Your manuscript is now with our production department. 

If your institution or institutions have a press office, please let them know about your upcoming paper now to help maximize its impact. If they'll be preparing press materials, please inform our press team within the next 48 hours. Your manuscript will remain under strict press embargo until 2 pm Eastern Time on the date of publication. For more information please contact onepress@plos.org.

If we can help with anything else, please email us at plosone@plos.org. 

Thank you for submitting your work to PLOS ONE and supporting open access. 

Kind regards, 

PLOS ONE Editorial Office Staff

on behalf of

Dr. Arunava Roy 

Academic Editor

PLOS ONE
